# 3D Liquid Crystal Framework Enclosing Dual Compartments

**DOI:** 10.1002/anie.7487332

**Published:** 2026-08-26

**Authors:** Yang‐yang Zhao, Huan‐jun Lu, Helgard Ebert, Xiang‐bing Zeng, Chunlan Ma, Feng Liu, Carsten Tschierske, Goran Ungar

**Affiliations:** ^1^ Shaanxi International Research Center for Soft Matter State Key Laboratory for Mechanical Behavior of Materials Xi'an Jiaotong University Xi'an China; ^2^ Advanced Technology Research Institute of Taihu Photon Center School of Physical Science and Technology Suzhou University of Science and Technology Suzhou China; ^3^ Department of Chemistry Martin Luther University Halle‐Wittenberg Halle Germany; ^4^ School of Chemical Materials and Biological Engineering University of Sheffield Sheffield UK; ^5^ Institute of New Concept Sensors and Molecular Materials Shaanxi Provincial Key Laboratory of New Concept Sensors and Molecular Materials Xi'an Jiaotong University Xi'an China

**Keywords:** bolapolyphiphiles, fluorous cages, GISAXS, electron density, martensitic transition, multicompartment structures, soft organic frameworks, supramolecular liquid crystals

## Abstract

We report a new 3D‐ordered liquid‐crystal (LC) phase where alkyl and fluoroalkyl side‐chains are confined within separate stretched octahedral cages of a tetragonal framework with struts of aromatic rods held together by their hydrogen‐bonded ends acting as flexible hinges. This is the first example of a multicolor micellar LC heralding the development of soft and dynamic dual‐compartment frameworks, also capable of a martensitic‐type phase transition.

## Graphical abstract

A new type of liquid crystal phase is reported, having the conjugated rod‐like molecular cores form a 3D framework of elongated octahedral cages filled alternatively with alkyl and fluoroalkyl pendant chains. The cages are held together by flexible H‐bonding hinges, making them adaptable to thermal expansion and stable in a wide temperature range. The anisotropic volume changes create morphology characteristic of a martensitic transition.

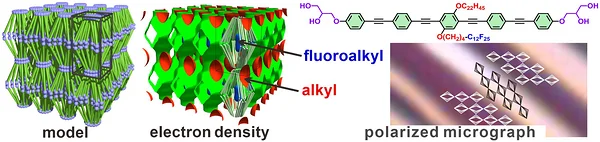

Self‐assembly in solution, on surfaces and in the solid‐state has been studied extensively [1, 2], but the understanding of complex structure formation in fluids is still at its infancy. Among them, liquid crystals (LCs) represent fluids or soft solids with a degree of long‐range order but without preferred positions of individual molecules, hence retaining molecular mobility [3]. Many liquid crystals consist of “hard” cores in the shape of columns, networks or spheres that are embedded in a continuum of the attached molten flexible chains [3]. Recently, a growing new class of LC phases with inverted structure has emerged, where soft domains of pendant flexible chains are framed by “hard” moieties, usually conjugated aromatic rods, referred to as LC honeycombs [4]. In these 2D ordered arrays infinitely long prismatic cells of different polygonal cross‐sections are separated by partition walls consisting of parallel rigid aromatic rod‐like cores of the constituent molecules. The rods are held together by their sticky ends, usually hydrogen‐bonding glycerol groups. The interior of the cells is filled by the flexible side‐chains. The constituent molecules may bear one side‐chain (T‐shaped) [4] or two chains at the same (π‐shaped) [5] or opposite sides (X‐shaped) [6].

When the two side‐chains of an X‐shaped molecule are incompatible, as e.g. one alkyl, the other perfluoroalkyl, polygonal honeycombs of two or more “colors” result, ranging from the simple “chessboard” 2‐color square honeycomb (Figure 1b) [6, 7] via the 2‐color kagome tiling combining hexagonal and triangular cells (Figure 1c) [8], to complex tilings [9, 10], where the unit cell contains up to 18 polygonal prismatic cells of five different shapes and compositions, considered a distorted quasicrystal approximant (Figure 1d) [9]. Related multicolor patterns were also found for star‐shaped molecules [11] and block copolymers [12, 13, 14].

**FIGURE 1:**
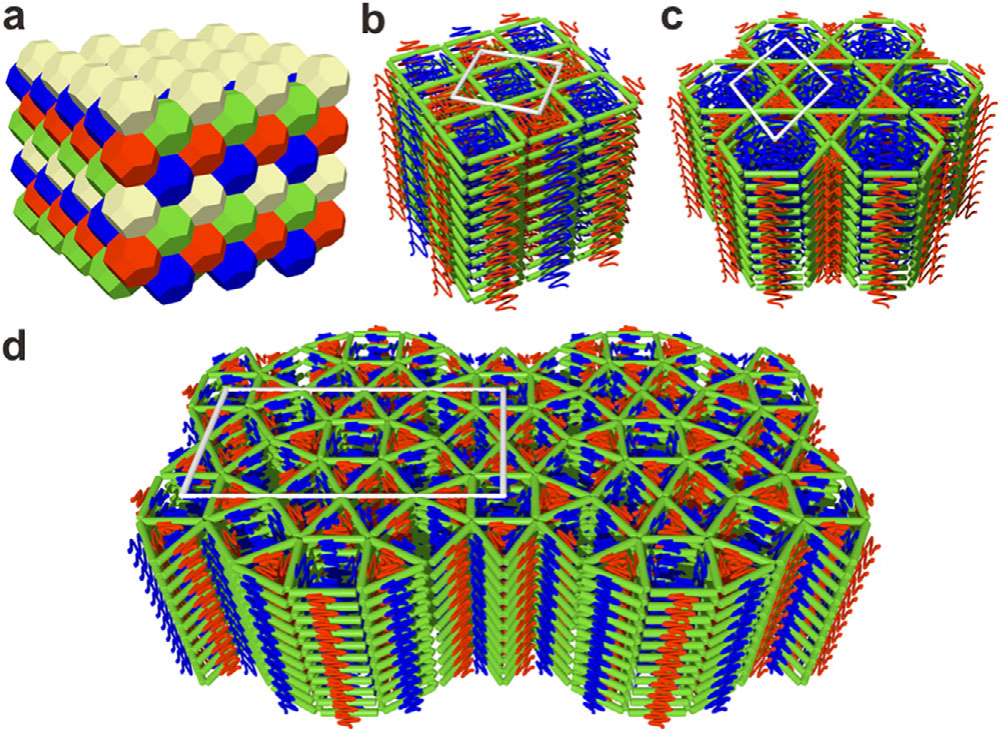
2D and 3D multicompartment multicolor soft matter. (a) Simulated structure of a block‐copolymer melt (re‐drawn after ref.19). (b–d) Inverse columnar “honeycomb” LCs. (b) square chessboard tiling [6]; (c) kagome tiling [8]; (d) complex distorted quasicrystal approximant [9].

In this work, we turn to soft mesophases with *polyhedral* cells, namely stretched octahedral compartments having edges of long rigid π‐conjugated rods and interiors alternatingly filled by the alkyl and fluoroalkyl side‐chains.

Though isolated supramolecular coordination cages with polyhedral shape and fluorous interior are known [15], attempts to produce coordination networks [16] or covalent organic frameworks representing periodic arrays of such alternating cells are still on the way [17, 18]. For block copolymers, “multicolored” polyhedral packings were predicted by simulations (Figure 1a) [19, 20].

Compound **1**, being the first forming a multicompartment cage structure in a liquid crystalline phase (Figure 2a) consists of a glycerol end‐capped oligo(*p*‐phenylene ethynylene) (OPE) core with five benzene rings, a semiperfluorinated (R_F_) and an n‐alkyl chain (R_H_) attached at opposite sides of the central ring. The choice of a polyfluorinated side‐chain was based on the unique property of R_F_‐chains to be both hydrophobic and lipophobic, thus tending to separate from both the hydrocarbon and the glycerol parts of our molecule.

**FIGURE 2:**
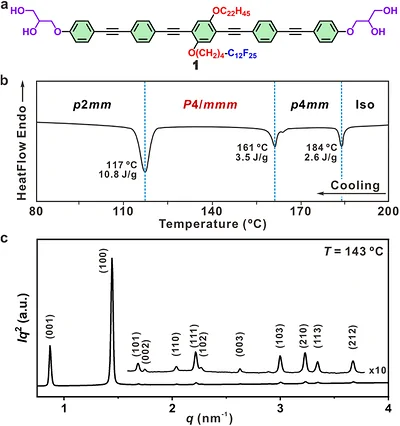
(a) Chemical structure of compound **1**. (b) Cooling DSC thermogram recorded at 10 K/min. Iso = melt, *p*4*mm* and *p*2*mm* = square and rectangular columnar phases, *P*4/*mmm* is the new 3D tetragonal phase. (c) SAXS powder diffractogram of the tetragonal LC phase.

Compound **1** was synthesized by repetitive Sonogashira cross‐coupling as key steps (see Section S1 with Scheme S1 and Figures S1–S4) and studied by powder small‐ and wide‐angle x‐ray scattering) (SAXS/WAXS), grazing incidence SAXS (GISAXS), calorimetry (DSC) and detailed polarized optical microscopy (POM). For details of synthesis and chemical and physical analysis, see Supporting Information.

The DSC cooling thermogram is shown in Figure 2b. The point of interest here is the phase with space group *P*4/*mmm*, spanning a 44K range between a square (*p*4*mm*) and a rectangular (*p*2*mm*) columnar phase. Regarding the adjacent columnar honeycombs, while they are not considered in this report, from our experience on similar compounds [6, 7] we can assume that the square columnar *p*4*mm* phase has weakly segregated R_H_ and R_F_ chains, while in the *p*2*mm* the two are separated more strongly. Both phases are two‐color chessboard‐type, with the cells likely to have square and rhombic cross‐sections, as squares had collapsed due to thermal shrinkage of the filler side‐chains. Powder SAXS patterns of the two columnar phases are shown in Figure S5.

Powder SAXS of the 161‐117°C phase is shown in Figure 2c. Its numerous Bragg peaks were successfully indexed on a tetragonal 3D lattice with *a* = 4.40 nm and *c* = 7.09 nm at 143°C. Indexing was helped by the GISAXS pattern of a surface‐aligned thin film (Figure 5a). LC nature of the phase is confirmed by the absence of Bragg peaks in WAXS; see Figure S6. The measured and calculated SAXS spacings and peak intensities are given in Table S1. As there are no systematic absences, space group *P*4*/mmm* (no. 123) was deduced. The electron density (ED) map, shown in Figure 3a, was calculated based on intensities of the four strongest Bragg reflections. For details, see Supporting Information. This is a new, previously unreported mesophase.

**FIGURE 3:**
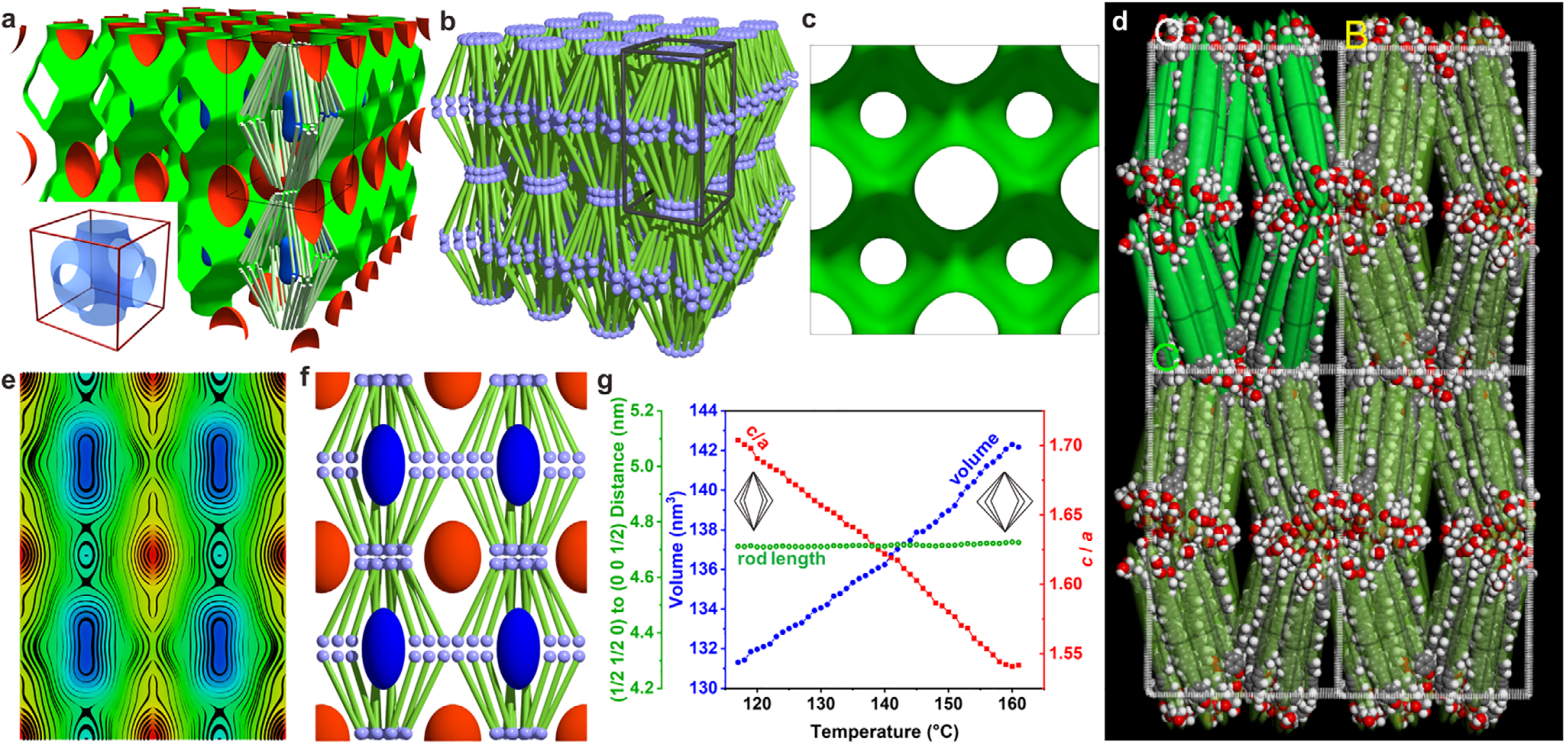
(a) Electron density map of the *P*4/*mmm* phase. (b) Schematic depiction of molecular arrangement; for color code see Figure 2a. (c) Top view of medium‐ED isoelectron surface; larger hole is between R_H_ domains. (d) Energy‐minimized molecular model of 4 unit cells without side‐chains; OPE cores are enveloped in best‐fitting ellipsoids. (e) ED map of the diagonal (110) plane. Blue (maxima), red (minima) and green (medium) correspond to R_F,_ R_H_ and OPE domains, respectively. (f) Schematic structure of (110) plane. (g) *T*‐dependence of unit cell volume (blue), *c/a* ratio (red) and (0 1/2 1/2) to (1/2 0 0) distance (effective rod length, green).

In each unit cell there is a strong ED maximum (blue) indicating the location of the electron‐rich fluorocarbon R_F_ chains, and a deep minimum (red) coming from the low‐density aliphatic R_H_ chains. The intermediate density (green) region is a continuum interposed between the two ED extremes. It represents spaces containing the π‐conjugated OPE cores, glycerol end‐groups, and possibly regions with mixed R_F_ and R_H_ chains; all have similar ED.

The green surface is a rough approximation of stretched minimum curvature Schwarz P‐surface [21], normally separating constituent networks of the bicontinuous cubic Plumber's Nightmare (PN) phase (inset in Figure 3a). The *P*4*/mmm* structure could be considered a distorted alternating‐network PN. PN is well‐known in lyotropic LCs [22], but very rare in thermotropics [23] and block‐copolymers [24]. However, in our case the surface dividing the R_H_ and R_F_ regions is significantly modified (Figure S7), containing straight rods with the glycerol end‐groups which form oblate spheroids or disks. Each spheroid contains ∼63 glycerols, being the number of molecules in a unit cell (see calculation in Section S5). The π‐conjugated rods associate in bundles of ∼8 parallel molecules fused by the hydrogen‐bonding glycerol domains forming stretched octahedral cells (Figure 3b).

The distance from the center of the square base of the unit cell (½ ½ 0) to the middle of the vertical edge (0 0 ½) is 4.7 nm, that is, only slightly longer than the molecular core (glycerol‐to‐glycerol ends 4.4 nm, Figure S8). This is consistent with the model in Figure 3.

The models in Figure 3b–f illustrate the structure of the *P*4/*mmm* phase. Its versatility is apparent: The H‐bonded glycerols act as flexible hinges, allowing the octahedral cells to adjust to changes in total volume of their content. Thus, by contracting laterally by 10.9% in area and extending longitudinally by 4.0% along z, the structure is seen to adjust to thermal contraction of R_H_/R_F_ chains on cooling by 7.2% between 161°C and 117°C like a 3D pantograph (Figure 3g). This makes the *P*4/*mmm* phase stable in the T‐range of 44 K. Linear extrapolation gives a cubic structure (*c/a* = 1) at *T* = 297°C, with a = c = 5.59 nm, and the (½ ½ 0) to (0 0 ½) distance 4.8 nm, only slightly larger than the molecular length. In fact, a single‐color body‐centered cubic phase with an octahedral network and a very similar lattice parameter (*a*_cub_ = 5.40 nm) has been found for an X‐shaped compound with the same OPE core but with side‐groups equal to each other, both branched alkyl (compound 2, Figure S9) [25]. The (½ ½ 0) to (0 0 ½) distance was 4.7 nm, almost the same as found for the P4/mmm phase in 1. Thus, the P4/mmm phase is considered a stretched R_H_/R_F_ segregated two‐colour version of the Im3¯m cubic phase in 2.

The distortion of the framework in the current *P*4*/mmm* phase relative to that in the single‐color phase of **2** is also in that the two octahedral cages in the unit cell are now of unequal volume, the R_F_ cage being smaller than the R_H_ one. This assumption is based on the fact that in the high‐T square chessboard the R_H_ and R_F_ volumes were similar, while on cooling R_F_ shrinks more than R_H_ (thermal expansivities are 1.7 × 10^−3^ and 1.1 × 10^−3^ K^−1^ for *n*‐C_8_F_18_ and *n*‐C_8_H_18_, respectively [26]). The difference in cage volumes is obvious from the ED map in Figure 3c. The steeper shrinkage of R_F_ is consistent with straightening and bundle formation of R_F_ chains due to some fluorophilic aggregation—note the more elongated high‐ED (blue) ellipsoids in Figure 3e.

Gradual increase of R_F_/R_H_ chain separation is visible in Figure S6 by the appearance of a separate high‐*q* shoulder (spacing 0.45 nm) attributed to inter‐chain correlation of alkyls. WAXS is otherwise dominated by the electron‐rich fluorocarbons. Note that the R_F_ WAXS peak around *q* = 12 nm is gradually shifting to higher *q* on cooling due to R_F_ shrinkage even though one would expect a low‐*q* shift based purely on increasing phase separation.

Actually, there is a series of related single‐color LC network structures. These are shown in Figure 4a–c with decreasing relative side‐chain volume, increasing junction valency *N*, and decreasing angle α between molecular bundles (struts). The node valences of the three frameworks are *N* = 6 [27], 8 [25], and 12–14 [28]. The respective structures are based on square, bent square and triangular meshes. Square meshes (windows, α = 90°) are sides of primitive cubes of the “single Plumbers Nightmare” network (Figure 4a) [27]. Bent square and bent rhomboid meshes constitute the open octahedra in the cubic ($Im\bar{3}m$) phase (*α* = 70°, Figure 4b) [25] and in the current tetragonal *P*4/*mmm* phase. Triangular windows (meshes, *α* = 60°) are the sides of distorted tetrahedral cells of the A15 Frank‐Kasper type network (Figure 4c) [28]. Compared to the α = 70° in the body‐centered cubic lattice in Figure 4b, in the distorted 2‐color *P*4*/mmm* version the mesh angles are 37° (top) and 125° (side) at 143°C, with *V* ^1/3^/*L* = 0.31.

**FIGURE 4:**
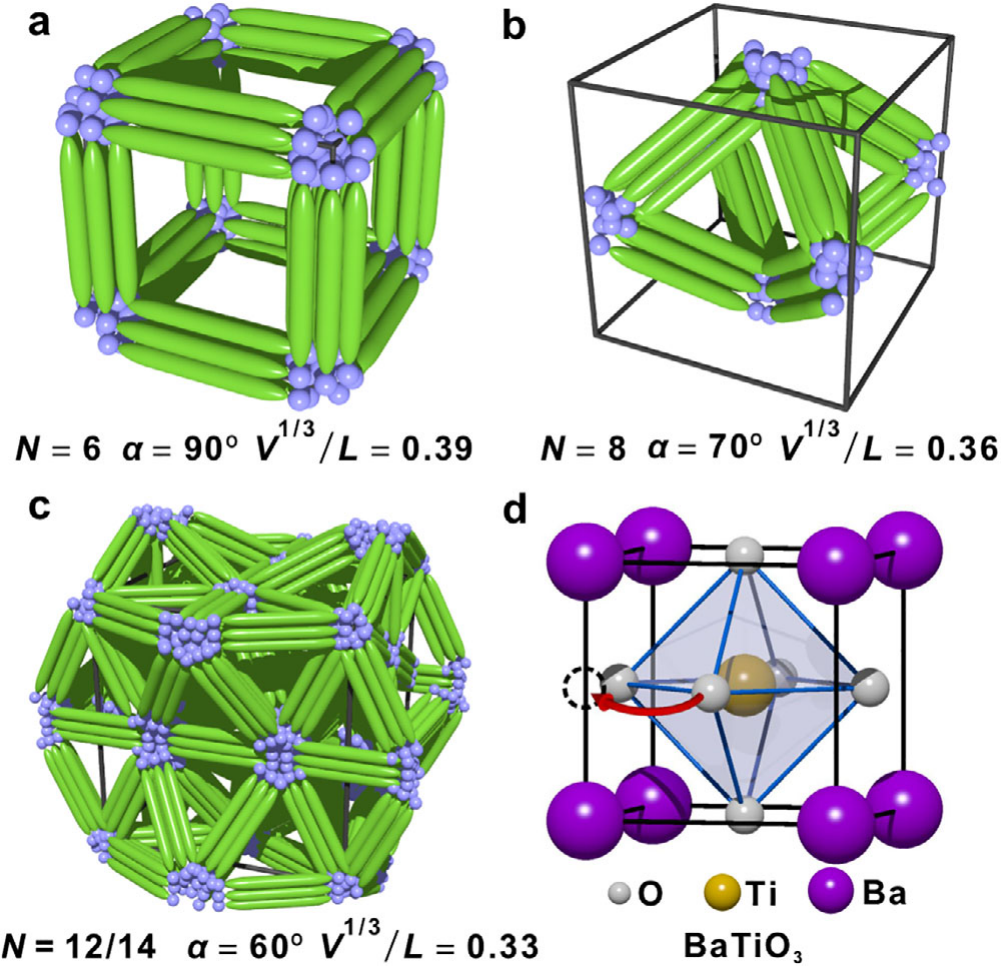
(a–c) Rod‐bundle packing in three cubic LC phases of X‐shaped molecules with decreasing volume‐to‐length *V* ^1/3^ */L* ratio: (a) primitive $Pm\bar{3}m$ [27], (b) body‐centered $Im\bar{3}m$ in Compound 2 [25], (c) A15 $Pm\bar{3}n$ phase [28]. Values of *V^1/3^/L* and node valency *N* are given below the models. (d) Unit cell of tetragonal BaTiO_3_ perovskite; compare with (b).

A relationship can also be drawn between structures of compounds **1** and **2**, and that of inorganic perovskites, as illustrated in Figure 4d. The analogy can be made symbolically to the tetragonal perovskite phase of, for example, BaTiO_3_, if Ba, Ti and O atoms are replaced, respectively, by R_H_, R_F_ and glycerol domains; the octahedral oxygen cage is then rotated by 45° around the vertical axis (see arrow) and expanded somewhat laterally. This brings the oxygens (viz., glycerols) from face‐centers in perovskite to edge‐centers in **1** and **2**.

GISAXS pattern of a thin film of **1**, recorded in the *P*4/*mmm* phase, is shown in Figure 5a. In addition to helping index the Bragg reflections, the pattern gives information about the LC alignment relative to the substrate surface. Three types of orientation are seen, shown in Figure 5b; the base vectors of their respective reciprocal lattices are shown on the GISAXS pattern with three different colors. The unit cell is either on its side, that is, on a (100) plane (red), on its square base (001, green), or on its diagonal (101) plane (yellow). The (101) orientation helps confirm the proposed structure since the framework prefers to anchor to the substrate with one of its densely packed planes. In the (101) case, it is parallel to the tilted OPE rods, as shown in Figure 5b. In all three orientations, the tendency is evident for the glycerol groups to be in contact with the polar oxidized Si surface.

**FIGURE 5:**
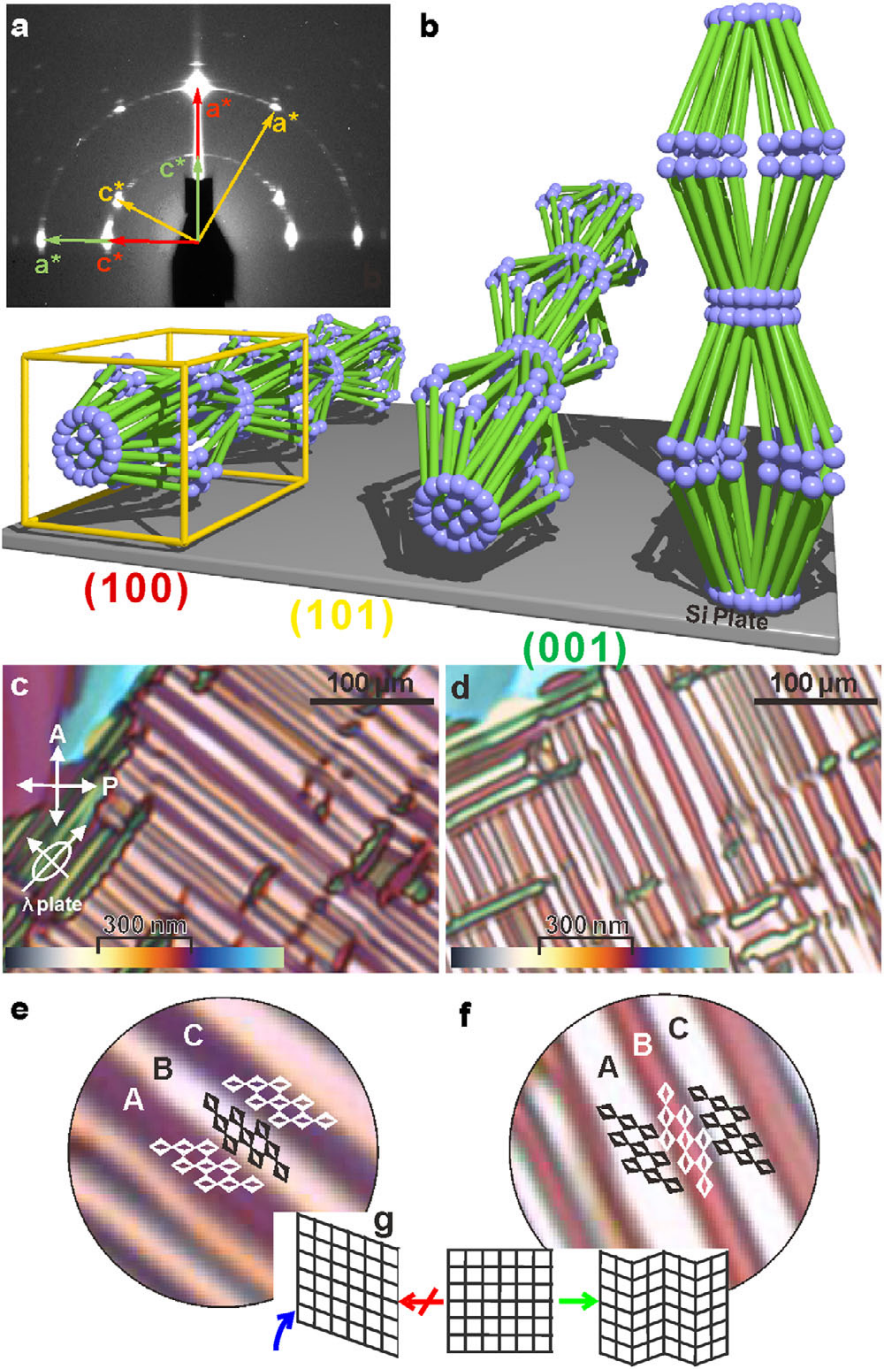
(a) GISAX pattern from a thin film of 1 in the tetragonal phase, and (b) models of the octahedral cages with Miller indices of the planes facing the substrate. The GISAXS pattern shows three orientations, colored red, green, and yellow, with the index of the lattice plane facing the substrate indicated. (c, d) Optical micrographs, between crossed polarizers and a 530 nm *λ*‐plate, of the same area in the tetragonal phase, rotated in‐plane by 27°. Orientation of polarizers and *λ*‐plate indicatrix are shown on the left. The difference in optical retardation between the stripes is indicated on the Michel‐Levy chart at the bottom of the micrographs. Red means zero retardation (*Γ* = 0) between the e and o ray in the sample; blue‐green means *Γ*>0 (c‐axis oriented NE‐SW), and yellow‐white means *Γ*<0 (c‐axis NW‐SE). Δ*Γ*≈300 nm between stripes. (e, f) Zoomed‐in portions of (c) and (d) with added schematic strings of octahedral cages along the c‐axis. (g) Scheme illustrating how deformation of the high‐T square lattice on cooling splits it into stripes (right) and avoids mass material movement (blue arrow on left).

Polarized optical micrographs of the planar (100)‐oriented tetragonal phase of **1** taken with a λ‐retarder are shown in Figure 5c,d at two orientations. The orientation of the octahedral cells is shown in the enlarged details in Figure 5e,f. In magenta‐red stripes, the optic axis, being the slow axis and the crystallographic *c*‐axis, is parallel to one of the polarizers, while in white stripes it is oriented NW‐SE. As between the two images the sample was rotated by 27°, it follows that the octahedral cells in neighboring stripes are at 90°−27° = 63° to each other. This suggests that the adjacent stripes are twins in epitaxial relationship, as arctan(*c/a*) ≅ 60° (Figure 3g).

The alternatively colored stripes form as a homeotropic square columnar monodomain transforms into planar *P*4/*mmm* phase and the fourfold axis turns from perpendicular to parallel to the film surface. In projection, the rhombs in Figure 3f form by distortion of the square columnar cells. The stripes are a result of material contraction on cooling, causing the transition to, and further shrinkage of, the *P*4*/mmm* phase along *a*, and extension along *c*. The stripes are a way of reducing the need for macroscopic material movement, in the diffusionless displacive transformation known as martensitic transition [29]. Two possible ways of deforming the square 2D lattice of the *p*4*mm* phase on cooling through the *P*4/*mmm* phase are depicted schematically in Figure 5g. Starting from the sketch in the middle, the path to the left would require long‐distance material movement (blue arrow). However, by splitting the square monodomain into stripes (path to the right) reduces material migration to only local.

It should be mentioned that martensitic transformation has been observed and studied in many different systems, first when austenite steel converts to martensite, forming characteristic shear stripes, and later in organic molecular crystals, colloids, and, in one case, in an LC blue phase [29, 30].

In summary, the first example of a multicolor micellar liquid crystal has been obtained and characterized, being the first soft matter system in which chemically different species (here R_F_/R_H_) are separated into a periodic array of alternating micellar aggregates embedded in polyhedral 3D cages. This complex structure, combined with the flexibility of the LC state, paves the way towards superstructural machines based on a combination of amphiphilic and reticular self‐assembly. The combination of recognition capability with the associated martensitic transition, pattern formation, and responsive behavior may lead to interesting applications as multifunctional materials.

## Author Contributions

**Yang‐yang Zhao**: investigation, visualization, writing – review and editing, formal analysis. **Huan‐jun Lu**: investigation, methodology, validation, visualization, formal analysis, data curation, funding acquisition, writing – review and editing, supervision. **Helgard Ebert**: investigation, methodology, formal analysis, data curation. **Xiang‐bing Zeng**: software, investigation, funding acquisition, methodology, visualization, data curation, supervision. **Chunlan Ma**: visualization, formal analysis. **Feng Liu**: investigation, funding acquisition, methodology, validation, formal analysis, supervision, resources. **Carsten Tschierske**: methodology, conceptualization, investigation, funding acquisition, validation, writing – review and editing, project administration, data curation, supervision, resources. **Goran Ungar**: conceptualization, funding acquisition, writing – original draft, methodology, validation, writing – review and editing, project administration, data curation, supervision, resources.

## Conflicts of Interest

The authors declare no conflicts of interest.

## Supporting information




**Supporting File**: anie74452‐sup‐0001‐SuppMat.pdf.

## Data Availability

The data that support the findings of this study are available in the Supporting Information of this article.

## References

[1] J.‐M. Lehn , “Towards Complex Matter: Supramolecular Chemistry and Self‐Organization,” European Review 17 (2009): 263–280, 10.1017/S1062798709000805. PMC122664 11929970

[2] D. B. Amabilino , D. K. Smith , and J. W. Steed , “Supramolecular Materials,” Chemical Society Reviews 46 (2017): 2404–2420, 10.1039/C7CS00163K. 28443937

[3] J. W. Goodby , P. J. Collings , T. Kato , et al., Handbook of Liquid Crystals, 2nd ed. (John Wiley & Sons, 2014), 10.1002/9783527671403.

[4] C. Tschierske , C. Nürnberger , H. Ebert , et al., “Complex Tiling Patterns in Liquid Crystals,” Interface Focus 2 (2011): 669–680, 10.1098/rsfs.2011.0087. PMC3438576 24098852

[5] S. Poppe , C. Chen , Y. Cao , F. Liu , and C. Tschierske , “From Columns to Networks: Search Toward the Elusive Single Gyroid With π‐Shaped Polyphilic Liquid Crystalline Block Molecules,” Small Science 5 (2025): 2500157, 10.1002/smsc.202500157. PMC12257908 40666048

[6] C. Nürnberger , H. Lu , X. Zeng , et al., “Soft Self‐Assembled Sub‐5 Nm Scale Chessboard and Snub‐Square Tilings With Oligo(para‐phenyleneethynylene) Rods,” Chemical Communications 55 (2019): 4154–4157, 10.1039/C9CC00494G. 30809626

[7] W. S. Fall , C. Nürnberger , X. Zeng , et al., “An Ising Transition of Chessboard Tilings in a Honeycomb Liquid Crystal,” Molecular Systems Design & Engineering 4 (2019): 396–406, 10.1039/C8ME00111A.

[8] B. Glettner , F. Liu , X. Zeng , et al., “Liquid‐Crystalline Kagome,” Angewandte Chemie International Edition 47 (2008): 9063–9066, 10.1002/anie.200802957. 18924222

[9] X. Zeng , R. Kieffer , B. Glettner , et al., “Complex Multicolor Tilings and Critical Phenomena in Tetraphilic Liquid Crystals,” Science 331 (2011): 1302–1306, 10.1126/science.1193052. 21393540

[10] F. Liu , R. Kieffer , X. Zeng , et al., “Arrays of Giant Octagonal and Square Cylinders by Liquid Crystalline Self‐Assembly of X‐Shaped Polyphilic Molecules,” Nature Communications 3 (2012): 1104, 10.1038/ncomms2096. 23033080

[11] L. de Campo , T. Varslot , M. J. Moghaddam , J. J. K. Kirkensgaard , K. Mortensen , and S. T. Hyde , “A Novel Lyotropic Liquid Crystal Formed by Triphilic Star‐Polyphiles: Hydrophilic/Oleophilic/Fluorophilic Rods Arranged in a 12.6.4. Tiling,” Physical Chemistry Chemical Physics 13 (2011): 3139–3152, 10.1039/c0cp01201g. 21103506

[12] Y. Matsushita , K. Hayashida , and A. Takano , “Jewelry Box of Morphologies With Mesoscopic Length Scales—ABC Star‐Shaped Terpolymers,” Macromolecular Rapid Communications 31 (2010): 1579–1587, 10.1002/marc.201000169. 21567569

[13] H. Hückstädt , A. Göpfert , and V. Abetz , “Synthesis and Morphology of ABC Heteroarm Star Terpolymers of Polystyrene, Polybutadiene and Poly(2‐vinylpyridine),” MacromolecuMlar Chemistry and Physics 201 (2000): 296–307, 10.1002/(SICI)1521-3935(20000201)201:3<296::AID-MACP296>3.0.CO;2-U.

[14] H. Lei , X.‐Y. Liu , Y. Wang , et al., “Precisely Constructing Superlattices of Soft Giant Molecules via Regulating Volume Asymmetry,” Journal of the American Chemical Society 146 (2024): 33403–33412, 10.1021/jacs.4c09089. 39614813

[15] S. Sato , J. Iida , K. Suzuki , M. Kawano , T. Ozeki , and M. Fujita , “Fluorous Nanodroplets Structurally Confined in an Organopalladium Sphere,” Science 313 (2006): 1273–1276, 10.1126/science.1129830. 16946067

[16] M. Jang , T. Yamaguchi , K. Ohara , M. Kawano , and M. Fujita , “Fluorous Pores in Coordination Networks,” Chemistry—An Asian Journal 4 (2009): 1524–1526, 10.1002/asia.200900293. 19739200

[17] W. Ji , P. Zhang , G. Feng , et al., “Synthesis of a Covalent Organic Framework With Hetero‐Environmental Pores and Its Medicine Co‐Delivery Application,” Nature Communications 14 (2023): 6049, 10.1038/s41467-023-41622-x. PMC10539374 37770448

[18] S. Bassoli , A. Krushelnitsky , F. Hamdi , et al., “Solid‐State NMR Reveals Mixed Side‐Chain Organization Across Pores in Amphiphilic Covalent Organic Frameworks,” Journal of Materials Chemistry A 14 (2026): 1647–1656, 10.1039/D5TA06558E.

[19] T. Dotera , “Cell Crystals: Kelvin's Polyhedra in Block Copolymer Melts,” Physical Review Letters 82 (1999): 105–108, 10.1103/PhysRevLett.82.105.

[20] L. Li , Z. Xu , and W. Li , “Emergence of Connected Binary Spherical Structures From the Self‐Assembly of an AB_2_C Four‐Arm Star Terpolymer,” Macromolecules 55 (2022): 9890–9899, 10.1021/acs.macromol.2c01919.

[21] A. L. Mackay , “Periodic Minimal Surfaces,” Nature 314 (1985): 604–606, 10.1038/314604a0.

[22] J. M. Seddon , “Lyotropic Phase Behaviour of Biological Amphiphiles,” Berichte der Bunsengesellschaft für physikalische Chemie 100 (1996): 380–393, 10.1002/bbpc.19961000324.

[23] Y.‐X. Li , R.‐Y. Jia , G. Ungar , et al., “Plumber's Nightmare″‐A Tight Liquid Organic Double Framework,” Angewandte Chemie International Edition 136 (2024): e202413215, 10.1002/ange.202413215. 39105624

[24] H. Lee , S. Kwon , J. Min , et al., “Thermodynamically Stable Plumber's Nightmare Structures in Block Copolymers,” Science 383 (2024): 70–76, 10.1126/science.adh0483. 38175890

[25] C. Chen , M. Poppe , S. Poppe , C. Tschierske , and F. Liu , “Liquid Organic Frameworks: A Liquid Crystalline 8‐Connected Network With Body‐Centered Cubic Symmetry,” Angewandte Chemie International Edition 132 (2020): 21006–21011, 10.1002/ange.202008784. PMC7693253 32692869

[26] M. Campos‐Vallette and G. Diaz‐Fleming , “Some Physical and Thermodynamic Properties of n‐CnF2n+2 Compounds With n = 4–8,” Semina Ciências Biológicas E Da Saúde 4 (1983): 405–409, 10.5433/1679-0367.1983v4n14p405.

[27] S. Poppe , X. Cheng , C. Chen , et al., “Liquid Organic Frameworks: The Single‐Network “Plumber's Nightmare” Bicontinuous Cubic Liquid Crystal,” Journal of the American Chemical Society 142 (2020): 3296–3300, 10.1021/jacs.9b11073. 32020802

[28] C. Chen , M. Poppe , S. Poppe , M. Wagner , C. Tschierske , and F. Liu , “Tetrahedral Liquid‐Crystalline Networks: An A15‐Like Frank–Kasper Phase Based on Rod‐Packing,” Angewandte Chemie International Edition 134 (2022): e202203447, 10.1002/ange.202203447. PMC9321821 35470526

[29] S. K. Park and Y. Diao , “Martensitic Transition in Molecular Crystals for Dynamic Functional Materials,” Chemical Society Reviews 49 (2020): 8287–8314, 10.1039/D0CS00638F. 33021272

[30] H. M. Jin , X. Li , J. A. Dolan , et al., “Soft Crystal Martensites: An In Situ Resonant Soft X‐Ray Scattering Study of a Liquid Crystal Martensitic Transformation,” Science Advances 6 (2020): eaay5986, 10.1126/sciadv.aay5986. PMC7101220 32258402

