# Supplementary material for: 3D Liquid Crystal Framework Enclosing Dual Compartments
Source: Angew Chem Int Ed Engl. 2026 Aug 26;65(41):e7487332. doi: 10.1002/anie.7487332 (PMC13638091; doi:10.1002/anie.7487332)
Supplement: Supplementary file 1 [file ANIE-65-e7487332-s001.pdf]

Supporting Information

3D Liquid Crystal Framework Enclosing Dual Compartments

Yang-yang Zhao,[1] Huan-jun Lu,[2] Helgard Ebert,[3] Xiang-bing Zeng,[4] Chunlan Ma,[2] Feng Liu,[1,5] Carsten Tschierske,[3] Goran Ungar[1,4]

1. Shaanxi International Research Center for Soft Matter, State Key Laboratory for Mechanical Behavior of Materials, Xi'an Jiaotong University, Xi'an 710049, China

2. Advanced Technology Research Institute of Taihu Photon Center, School of Physical Science and Technology, Suzhou University of Science and Technology, Suzhou 215009, China

3. Department of Chemistry, Martin Luther University Halle-Wittenberg, 06120 Halle, Germany

4. School of Chemical, Materials and Biological Engineering, University of Sheffield, Sheffield S1 3JD, U.K.

5. Institute of New Concept Sensors and Molecular Materials, Shaanxi Key Laboratory of New Concept Sensors and Molecular Materials, Xi'an Jiaotong University, Xi'an 710049, China

Correspondence: Huan-jun Lu (luhuanjun@usts.edu.cn); Goran Ungar (g.ungar@sheffield.ac.uk); Carsten Tschierske (carsten.tschierske@chemie.uni-halle.de)

Contents

1. Synthesis and Analytical Data 2
2. Phase Characterization Methods 7
3. Additional X-ray diffraction data 8
4. Electron density reconstruction 10
5. Molecular length and number of molecules in a unit cell 11
6. Additional figures 12
7. References 12

1. Synthesis and Analytical Data

1.1 General remarks

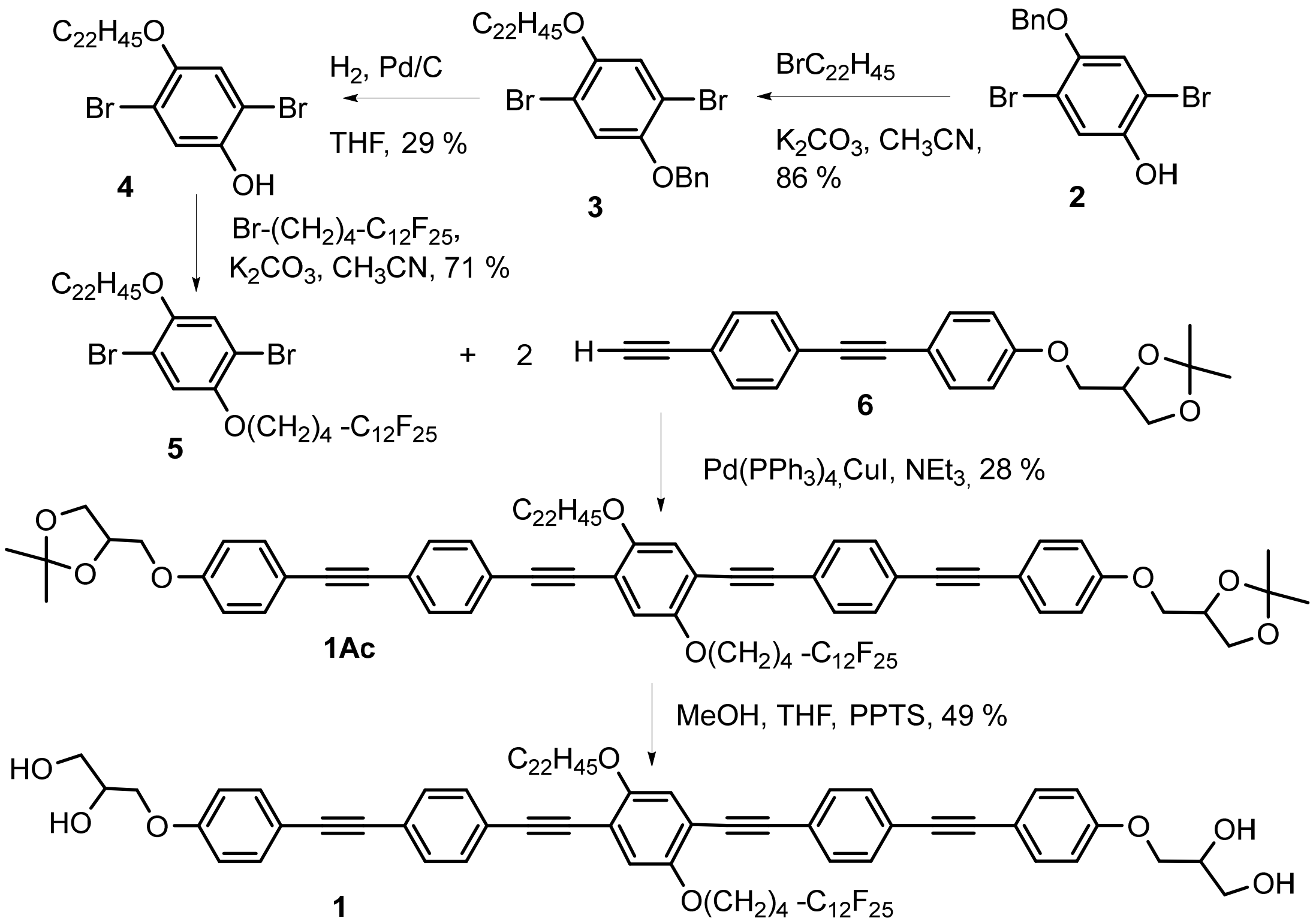

Scheme S1. Synthesis of compound **1.**

The synthesis of compound **1** was performed as outlined in Scheme S1. 5,5,6,6,7,7,8,8,9,9,10,10,11,11,12,12,13,13,14,14,15,15,16,16,16-Pentacosafluorhexadecylbromide was synthesized as reported in Ref. S1. 4-Benzyloxy-2,5-dibromophenol (**2**) and 3-*O*-[4-(4-ethynylphenylethynyl)phenyl]-1,2-*O*-isopropylideneglycerol (**6**) were prepared as described in Refs. S2 and S3 respectively. Because racemic 1,2-O-isopropylideneglycerol was used as starting material for the synthesis of **1** it represents a racemic mixture of 2 diastereomers. Triethylamine was distilled from CaH_2 and stored over molecular sieve. Dry acetonitrile was stored over molecular sieve. All other solvents were distilled prior to use and the building blocks, reagents and catalysts were used as received without further purification.

The purity was checked by thin-layer chromatography (TLC, silica gel 60 F254, Merck). Column chromatography was performed with silica gel 60 (0.063-0.2, Merck). ^{1}H-, ^{13}C- and ^{19}F-NMR spectra (Varian Unity 400/500 spectrometer) were recorded in $CDCl_3$ or pyridine-d_5 solutions, with tetramethylsilane as internal standard. All measurements were operated at 27 °C. Elemental analysis was not possible due to the high fluorine content. Mass spectra were recorded with a Bruker HR-ESI-TOF. The measurements were performed in negative mode in THF (1mg/mL) with 0.1 mg/mL LiCl.

1.2 Preparation of the intermediates

1-Benzyloxy-2,5-dibromo-4-docosyloxybenzene (3)

A mixture of 4-benzyloxy-2,5-dibromophenol (**2**, 2.00 g, 5.59 mmol), 1-bromodocosane (2.39 g 6.14 mmol), K_2CO_3 (3.9 g, 28 mmol) and Bu_4NI (tip of a spatula) in anhydrous acetonitrile (50 mL) was stirred for 12 h under reflux. After cooling to room temperature, the reaction was poured into water (100 mL) and the aqueous layer was extracted with Et_2O (3x50 mL). The combined organic layers were washed with water, saturated aqu. LiCl, water and brine (50 mL each). After drying over anhydrous Na_2SO_4, filtration and evaporation of the solvent, the crude product was purified by crystallization from petroleum ether. Yield: 3.22 g (86 %) colorless solid (m.p. 70-72 °C); ^{1}H NMR (400 MHz, $CDCl_3$) δ = 7.48 – 7.27 (m, 5H, Ar-*H*), 7.14 (s, 1H, Ar-*H*), 7.08 (s, 1H, Ar-*H*), 5.05 (s, 2H, OCH_2Ph), 3.93 (t, ^3J = 6.5 Hz, 2H, OCH_2), 1.85 – 1.72 (m, 2H, OCH$_2$CH_2), 1.51 – 1.39 (m, 2H, OCH$_2$CH$_2$CH_2), 1.37 – 1.16 (m, 36H, CH$_2$), 0.86 (t, *J* = 6.8 Hz, 3H, CH_3).

2,5-Dibromo-4-docosyloxyphenol (4)

3 (2.00 g, 3.00 mmol) was dissolved in THF (100 mL) and degassed. Pd/C (10 wt % Pd, 0.3 g) was added and after flushing with hydrogen the mixture was shaken under hydrogen atmosphere (30 psi) in a Parr hydrogenation apparatus for 24 hours at 25 °C. Afterwards the mixture was filtered through celite and the solvent was removed under reduced pressure in a rotatory evaporator. The residue was purified by column chromatography (eluent: $CHCl_3$) and crystallized from *n*-hexane. Yield: 0.51 g (29 %) colorless solid (m. p. 78-80 °C); ^{1}H NMR (400 MHz, $CDCl_3$) δ = 7.22 (s, 1H, Ar-*H*), 6.96 (s, 1H, Ar-*H*), 5.09 (s, 1H, O*H*), 3.91 (t, *J* = 6.5 Hz, 2H, OCH_2), 1.87 – 1.68 (m, 2H, OCH$_2$CH_2), 1.50 – 1.40 (m, 2H, OCH$_2$CH$_2$CH_2), 1.39 – 1.20 (m, 36H, CH_2), 0.86 (t, *J* = 6.8 Hz, 3H, CH_3).

1,4-Dibromo-5-docosyloxy-2-(5,5,6,6,7,7,8,8,9,9,10,10,11,11,12,12,13,13,-14,14,15,15,16,16,16-pentacosafluorohexadecyloxy)benzene (5)

A mixture of **4** (270 mg, 0.47 mmol) 5,5,6,6,7,7,8,8,9,9,10,10,11,11,12,12,13,13,14,14,15,15,16,16,16-pentacosafluorohexadecylbromide (354 mg, 0.47 mmol), K_2CO_3 (323 mg, 2.34 mmol) and Bu_4NI (tip of a spatula) in anhydrous acetonitrile (50 mL) was stirred for 8 h under reflux. After cooling to room temperature the mixture was poured into water (50 mL) and stirred for 30 min. The precipitated solid was sucked off and washed with water (100 mL). The crude product was dried at 25 °C and used without further purification for the next step. Yield: 420 mg (71 %) colorless solid (m.p. 104-108 °C); ^{1}H NMR (400 MHz, $CDCl_3$) δ = 7.07 (s, 2H, Ar-*H*), 3.97 (t, *J* = 5.6 Hz, 2H, OCH_2), 3.93 (t, *J* = 6.5 Hz, 2H, OCH_2), 2.27 – 2.11 (m, 2H, CH_2CF$_2$), 1.94 – 1.82 (m, 4H, OCH$_2$CH_2), 1.82 – 1.73 (m, 2H, CH_2CH$_2$CF$_2$),

1.50 – 1.39 (m, 2H, $OCH_2CH_2CH_2$), 1.38 – 1.19 (m, 36H, CH_2), 0.86 (t, *J* = 6.9 Hz, 3H, CH_3).

5-Docosyloxy-2-(5,5,6,6,7,7,8,8,9,9,10,10,11,11,12,12,13,13,14,14,15,15,-16,16,16-pentacosafluorohexadecyloxy)-1,4-bis(4-{4-[(2,2-dimethyl-1,3-dioxolane-4-yl)methoxy]-phenylethynyl}phenylethynyl)benzene (1Ac)

A mixture of **5** (420 mg, 0.34 mmol) and **6** (279 mg, 0.84 mmol) were dissolved in purified Et_3N (30 mL). After degassing with argon for 30 min [$Pd(PPh_3)_4$] (12 mg, 0.010 mmol) and CuI (1 mg, 0.007 mmol) were added and the mixture was refluxed for 7 h. After removing the solvent under vacuum the obtained residue was dissolved in $CHCl_3$/water (50 mL, each). The organic layer was separated and the aqueous layer was extracted twice with $CHCl_3$ (50 mL, each). The combined organic layers were washed with water, brine (50 mL, each) and dried over Na_2SO_4. After evaporation of the solvent the residue was purified by column chromatography (eluent: CH_2Cl_2) and crystallization from CH_2Cl_2/Et_2O. Yield: 164 mg (28 % d. Th.), yellow solid (m.p. 158-161 °C); ^{1}H-NMR (400 MHz, $CDCl_3$) δ = 7.49 – 7.40 (m, 12H, Ar-*H*), 7.00 (s, 1H, Ar-*H*), 6.99 (s, 1H, Ar-*H*), 6.90 – 6.85 (m, 4H, Ar-*H*), 4.50 – 4.42 (m, 2H, C*H*), 4.16 (dd, *J* = 8.5, 6.4 Hz, 2H, OCH_2), 4.12 – 3.99 (m, 6H, CH_2O, OCH_2CH_2), 3.95 (ddd, *J* = 9.6, 5.9, 1.6 Hz, 2H, CH_2O), 3.89 (ddd, *J* = 8.5, 5.8, 1.0 Hz, 2H, OCH_2), 2.26 – 2.09 (m, 2H, CH_2CF_2), 2.00 – 1.78 (m, 6H, $CH_2CH_2CF_2$, OCH_2CH_2), 1.58 – 1.48 (m, 2H, $OCH_2CH_2CH_2$), 1.45 (s, 6H, acetonide-CH_3), 1.42 – 1.16 (m, 42H, CH_2, acetonide-CH_3), 0.86 (t, *J* = 6.9 Hz, 3H, CH_2CH_3)

1.3 Synthesis and analytical data of 5-docosyloxy-2-(5,5,6,6,7,7,8,8,9,9,10,10,11,11,12,12,-13,13,14,14,15,15,16,16,16-pentacosafluorohexadecyloxy)-1,4-bis{4-[4-(2,3-dihydroxy-propyloxy)phenylethinyl]phenylethinyl}benzene 1

A mixture of **1Ac** (162 mg, 0.092 mmol) and PPTS (tip of a spatula) was dissolved in THF/MeOH (1:1, 40 mL) and stirred at 60 °C for 3 days. After finishing the reaction (TLC) the solvent was removed and the residue solved in THF (10 mL). The residue (PPTS) is filtered off and the solvent is evaporated under vacuum. The residue is purified by preparative thin-layer chromatography (chromatotron, Harrison research, eluent: HPLC-grade ethyl acetate) and crystallization from THF/MeOH. Yield: 76 mg (49 %) yellow highly fluorescent solid; for transition temperatures, see Fig. 2b; ^{1}H NMR (500 MHz, Pyridine d_5) δ = 7.71 (m, 4H, Ar-*H*), 7.64 (m, 4H, Ar-*H*), 7.58 (m, 4H, Ar-*H*), 7.44 (s, 1H, Ar-*H*), 7.43 (s, 1H, Ar-*H*), 7.05 (m, 4H, Ar-*H*), 4.93 (broad, 8H, OH, H_2O) 4.55 – 4.33 (m, 6H, C*H*OH, OCH_2OH), 4.20 – 4.07 (m, 8H, CH_2OAr), 2.31 (m, 2H, CH_2CF_2), 1.93 (m, 4H, CH_2), 1.85 (m, 2H, CH_2), 1.58 (m, 2H, CH_2), 1.3-1.2 (m, 36H, CH_2), 0.82 (t, *J* = 7.0 Hz, 3H, CH_3); ^{13}C-NMR (126 MHz, pyridine-d_5) δ = 160.04 (2C), 154.25, 153.69 (C_{ar}-O), 133.35(2C), 133.31(2C), 131.79 (4C), 131.73(2C), 131.68(2C), 124.02, 123.97, 117.29, 117.24, 115.15(2C), 115.12(2C), 114.94(2C), 114.3(2C) (C_{ar}-H, C_{ar}-C); 95.25 (2C), 92.45, 92.39,

88.80, 88.57, 88.28, 88.22, (C≡C); 71.08 (2C), 70.83 (2C), 69.53, 68.81, 64.00 (2C), (OCH, OCH_2); 31.86 (CH_2), 30.48 (t, *C*H_2-CF_2), 29.76 + 29.74 (10C), 29.66, 29.43, 29.40, 29.34, 28.66, 26.19, 22.67, 17.42 (CH_2); 14.00 (CH_3); ^{19}F-NMR (188 MHz, $CDCl_3$) δ = -81.13 (t, ^3J(F,F) = 9.8 Hz, 3F, CF_3), -114.65 (s, 2F, CF_2CH_2), -122.05 (14F, CF_2), -123.03 (s, 2F, CF_2), -123.78 (s, 2F, CF_2), -126.44 (s, 2F, CF_2); HR-ESI-MS: *m/z* [*M*+Cl]$^-$ 1707.5300 (calc. 1707.5528)..

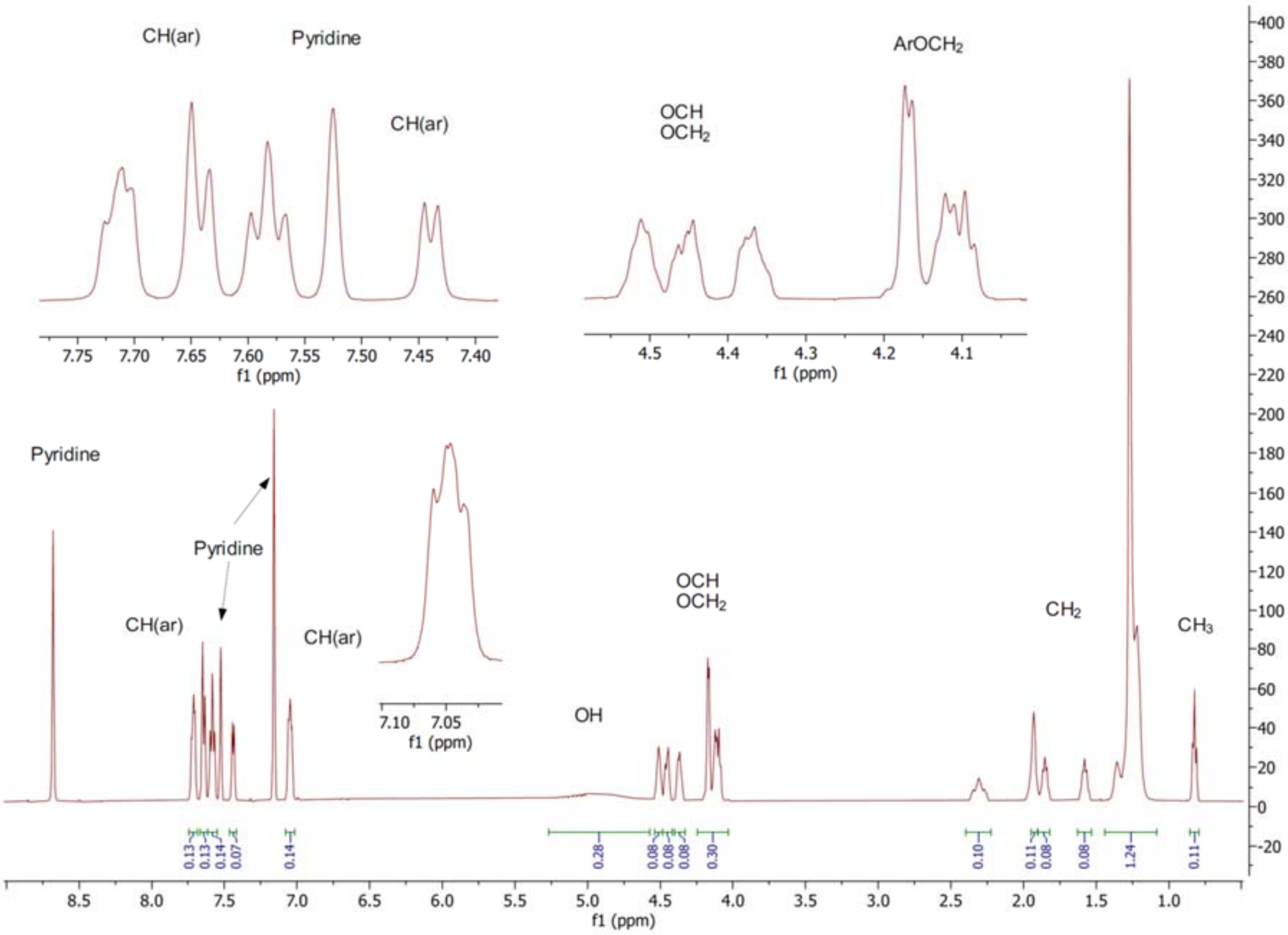

Figure S2. ^{1}H-NMR spectrum of compound **1** as measured at 27 °C in pyridine-d_6 (500 MHz).

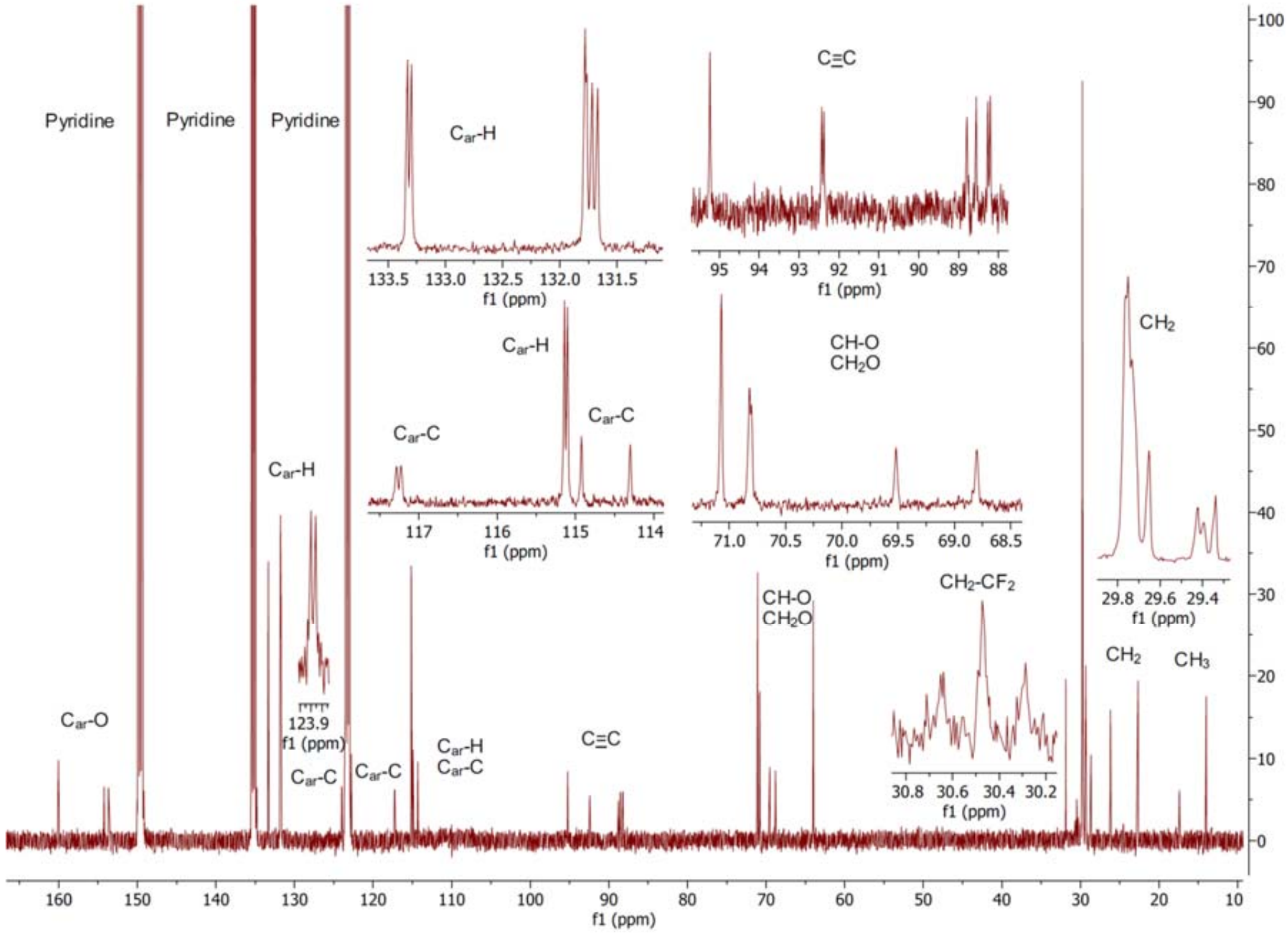

Figure S3. ^{13}C-NMR spectrum of compound **1** as measured at 27 °C in pyridine-d_6 (126 MHz).

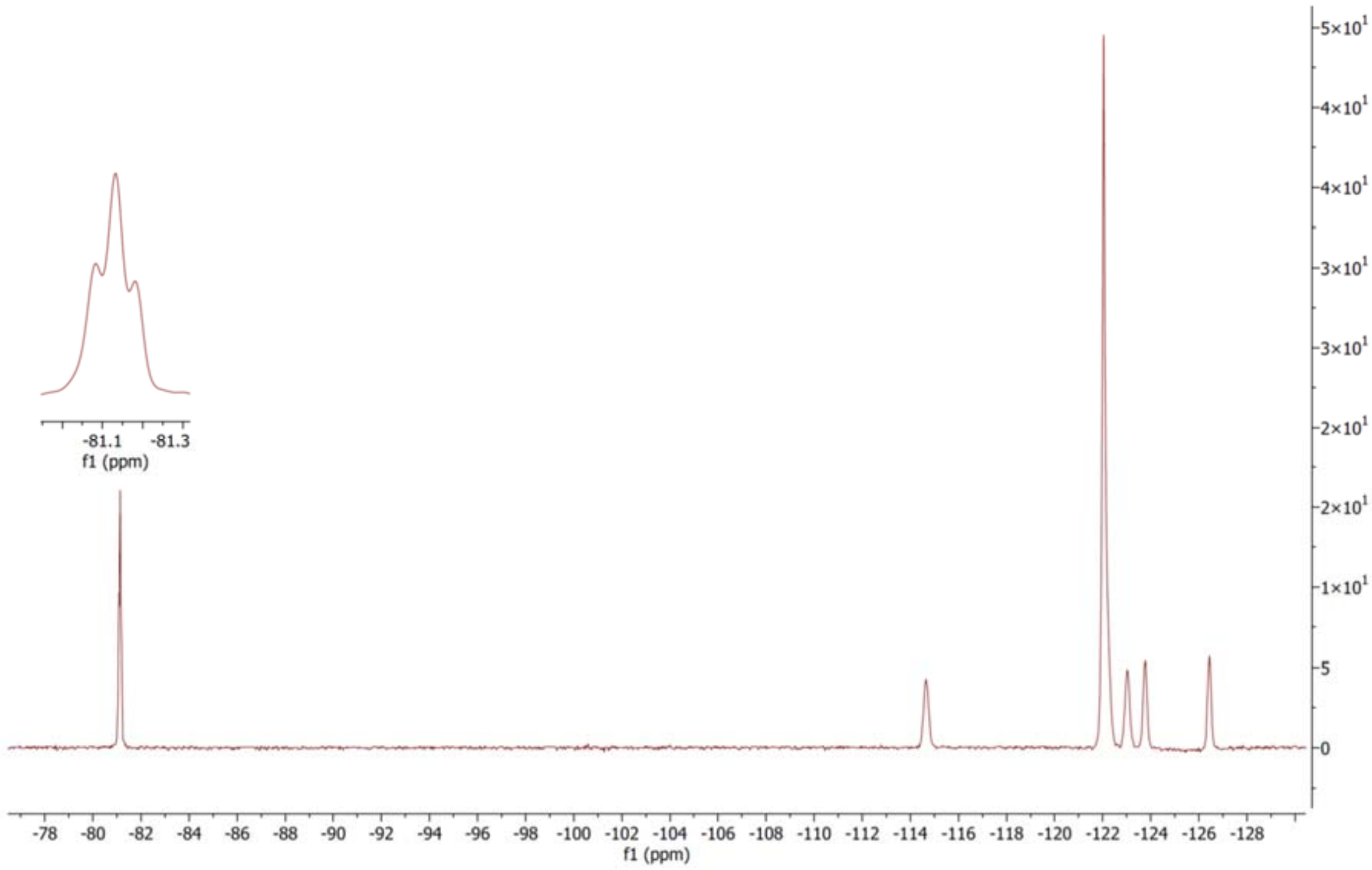

Figure S4. ^{19}F-NMR spectrum of compound **1** as measured at 27 °C in pyridine-d_6 (188 MHz).

2. Phase Characterization Methods

2.1 Differential scanning calorimetry

DSC thermograms were recorded on Perkin-Elmer DSC-7 instrument at heating and cooling rate of 10 K/min.

2.2 Polarized optical microscopy

Polarized optical microscopy was carried out on an Olympus BX50 microscope equipped with a Mettler FP85 hot stage. A 530 nm λ retarder plate was used. Optical retardation was measured with a Berek compensator.

2.3 X-ray diffraction

Small and Wide-angle X-ray diffraction patterns of powder-like samples (SAXS and WAXS) were recorded on Beamline I22 at Diamond Light Source, U.K. Samples were held in evacuated 1 mm capillaries. A modified Linkam hot stage with a thermal stability within 0.2 ºC was used, with a hole for the capillary drilled through the silver heating block and mica windows attached to it on each side. A Pilatus 2M detector was used.

Grazing incidence SAXS (GISAXS) experiments were carried out on beamline BM28 (XMaS) at European Synchrotron Radiation Facility (ESRF), France. Thin film samples were prepared by shearing on silicon substrate. The sample temperature was controlled by a custom-madr temperature-controlled stage. 2D diffractograms were collected using a MAR165 CCD camera.

Scattering vector q calibration and linearization were verified using several orders of layer reflections from silver behenate and n-tetracontane. Measurement of the positions and intensities of the diffraction peaks was carried out using Origin, where experimental diffractograms were fitted using Gaussian shaped peaks. The diffraction peaks were indexed on the basis of their peak positions and the lattice parameters, and the space groups were subsequently determined.

3. Additional X-ray diffraction data

Table S1 Experimental and calculated *d*-spacings of the observed SAXS reflections of the tetrangolar phase *P*4/*mmm* in compound **1** at 143 ºC. All intensities values are Lorentz and multiplicity corrected.

(*hkl*)	$d_{obs.}$ –spacing (nm)	$d_{calc.}$ –spacing (nm)	*intensity*	*phase*
(001)	7.08	7.09	77.7	0
(100)	4.39	4.40	100	0
(101)	3.74	3.74	1.74	/
(002)	3.55	3.55	0.461	/
(110)	3.11	3.11	0.268	/
(111)	2.85	2.85	2.29	π
(102)	2.77	2.76	0.885	/
(003)	2.37	2.36	0.781	/
(103)	2.08	2.08	7.31	0
(210)	1.96	1.97	2.06	/
(113)	1.89	1.88	1.61	/
(212)	1.72	1.72	2.14	/
(104)	1.64	1.64	0.164	/
(203)	1.61	1.61	0.226	/
(222)/(005)	1.42	1.42/1.42	0.719	/
(105)	1.35	1.35	0.317	/
(214)	1.32	1.32	0.358	/
(312)	1.29	1.30	0.438	/
(303)	1.25	1.25	0.0779	/
(006)	1.18	1.18	0.0619	/
a = 4.40 nm, *c* = 7.09 nm				

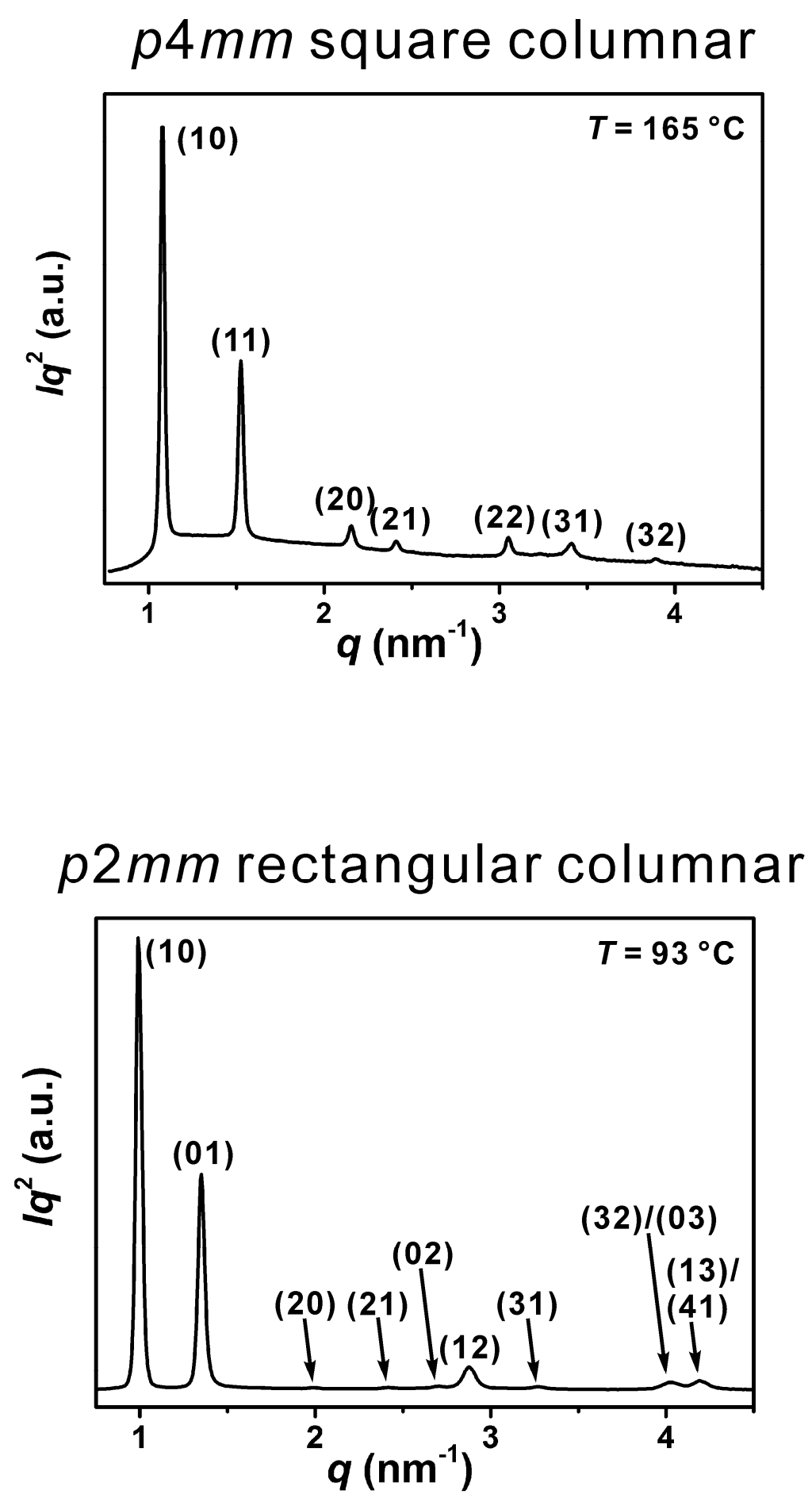

Figure S5. Powder SAXS traces of the two honeycomb columnar phases at temperatures above (top) and below (bottom) that of the 3D *P*4/*mmm* phase.

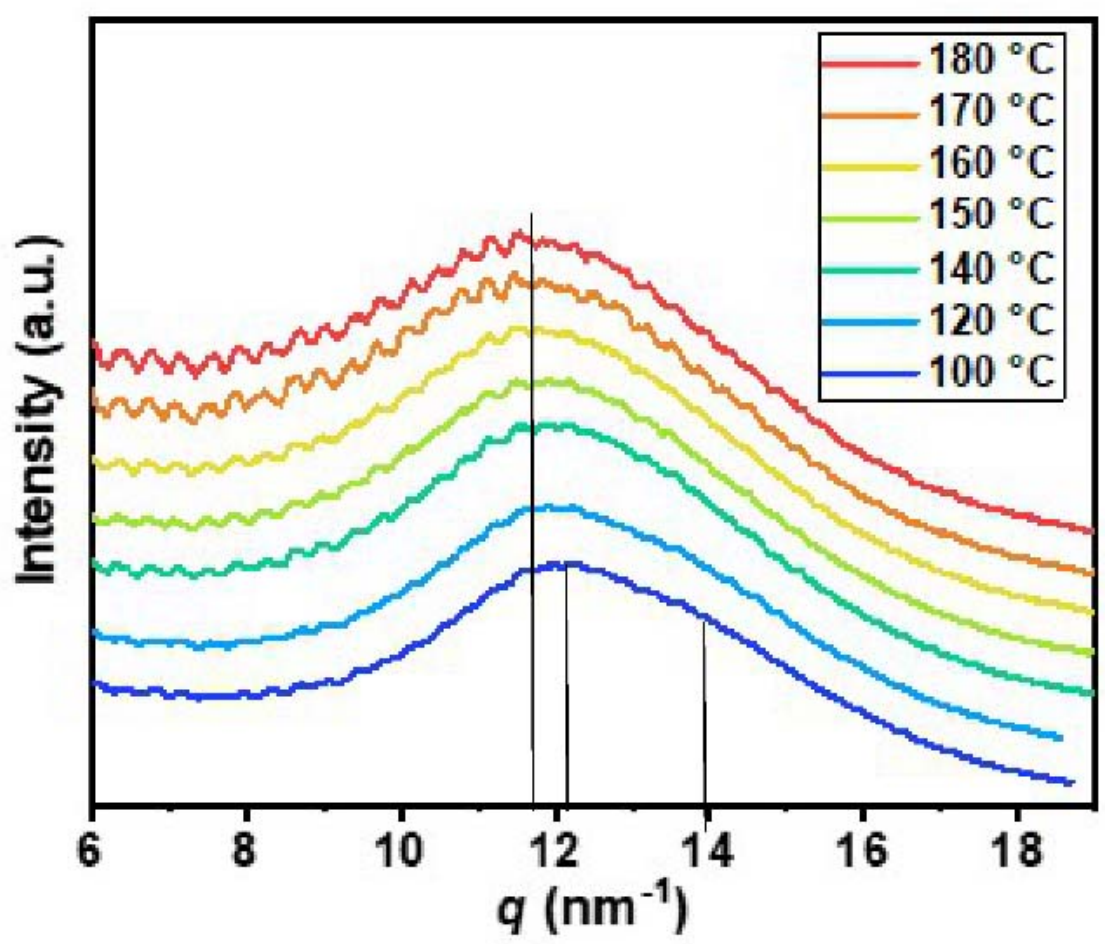

Figure S6. Temperature evolution of WAXS traces of compound **1** through *p*4*mm*, *P*4/*mmm* and *p*2*mm* phases.

4. Electron density reconstruction

Electron density maps could be reconstructed on the basis of the general formula

$$\rho(xyz) = \sum_{hkl} F(hkl)\exp[i2\pi(hx + ky + lz)] \quad \text{(Eqn. 1)}$$

Here $F(hkl)$ is the structure factor of a diffraction peak with index (hkl). It is normally a complex number and the experimentally observed diffraction intensity

$$I(hkl) = \mathrm{K} \bullet F(hkl) \bullet F^*(hkl) = \mathrm{K} \bullet |F(hkl)|^2 \quad \text{(Eqn. 2)}$$

Here K is a constant related to the sample volume, incident beam intensity etc. In this paper we are only interested in the relative electron densities, hence this constant is simply taken to be 1. Thus the electron density is

$$\rho(xyz) = \sum_{hkl} \mathrm{sqrt}[I(hkl)]\exp[i2\pi(hx + ky + lz) + \varphi_{hkl}] \quad \text{(Eqn. 3)}$$

As the observed diffraction intensity $I(hkl)$ is only related to the amplitude of the structure factor $|F(hkl)|$, the information about the phase of $F(hkl)$, φ_{hkl}, cannot be determined directly from experiment. However, the problem is significantly simplified with centrosymmetric structures, where the phase angle φ_{hkl} of a given (hkl) reflection was limited to 0 or π.

In the electron density map reconstruction of the *P*4/*mmm* phase, four strongest peaks (001), (100), (111) and (103) were used. As the phase angle of each diffraction peak can only be 0 (+) or π (-), there are 16 different phase combinations in total. Among the possible maps, half of them have reversed electron density of the others. Also, based on the criterion that the middle-density aromatic backbones form a continuous network in the middle of the unit cell, only 4 phase combinations (++++), (+++-), (++-+) and (++--) are left. An isoelectron surface formed by the OPE rods is shown for each map (Figure S4). Among them, the (++-+) combination was selected because it both conforms to the packing characteristics of rod-like molecules and exhibits a relatively smooth morphology.

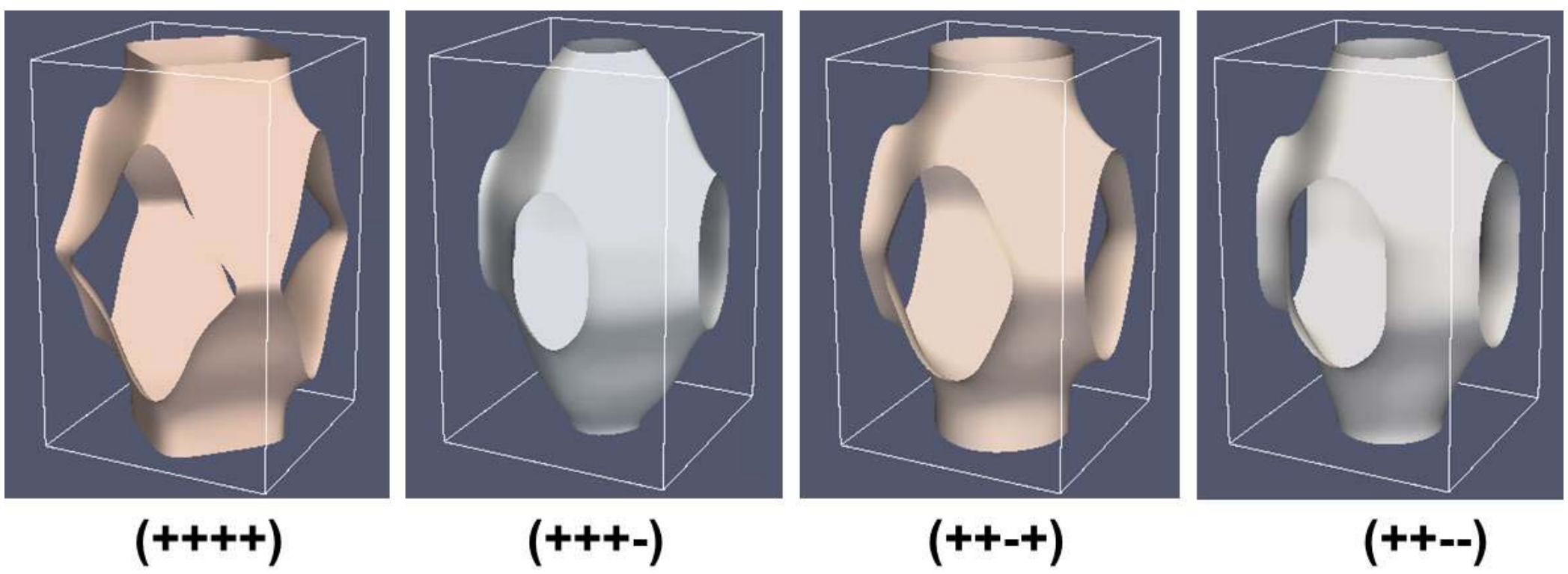

Figure S7. Reconstructed candidate electron density maps of the *P*4/*mmm* phase with different phase combinations.

5. Molecular length and number of molecules in a unit cell

Molecular length

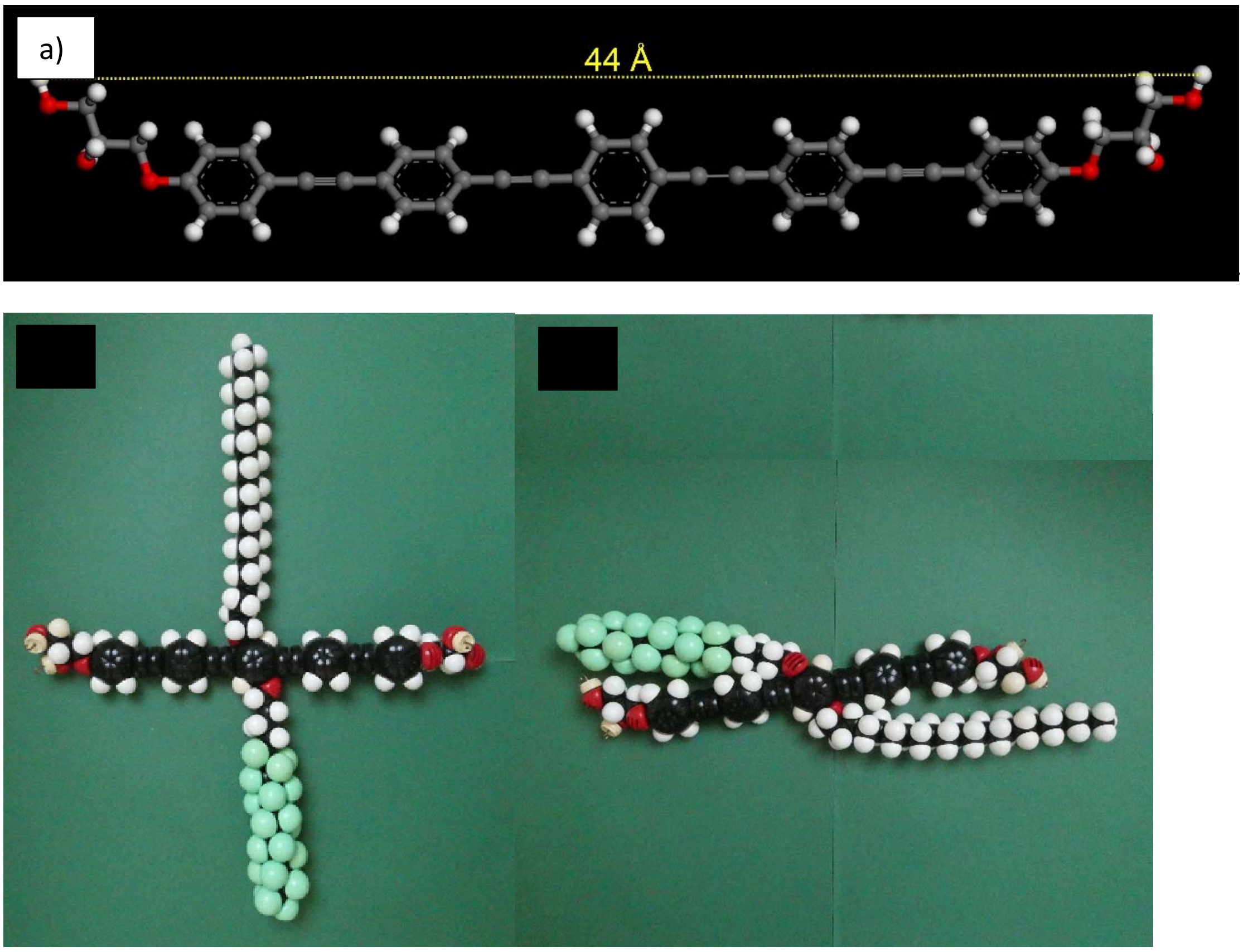

Figure S8. a) Model of the OPE core built in *Materials Studio*. The distance between the furthest H atoms of the glycerol groups was measured as molecular length; b, c) space filling CPK models of compound **1** showing b) the cross—shaped conformation and c) the conformation with chains almost parallel to the OPE core; the measured length between the furthest H atoms of the glycerol groups in CPK models is 4.4 nm).

Calculation of number of molecules

Unit cell volume:

$V_{cell} = a^2 * c = 4.40^2 * 7.09 = 137\ nm^3$

Molecular volume calculated with the crystal volume increments of Immirzi and Perini[S4]:

$V_{mol} = 1.95\ nm^3$

The Immirzi method assumes a crystal packing coefficient k=0.7. In comparison, k for liquids is typically 0.55. For liquid crystals we assumed k to be the average of the two, i.e. 0.63. Therefore the effective molecular volume in the LC phase becomes V_{mol} = 1.95*0.70/0.63 = 2.17 nm^3. So the number of molecules per unit cell is n_{cell} = 137/2.17 = 63.

Estimated number of molecules in each of the 8 bundles of OPE rods in the *P*4/*mmm* unit cell:

$n_{bundle} = n_{cell}/8 = 63/8 \approx 8$

6. Additional figures

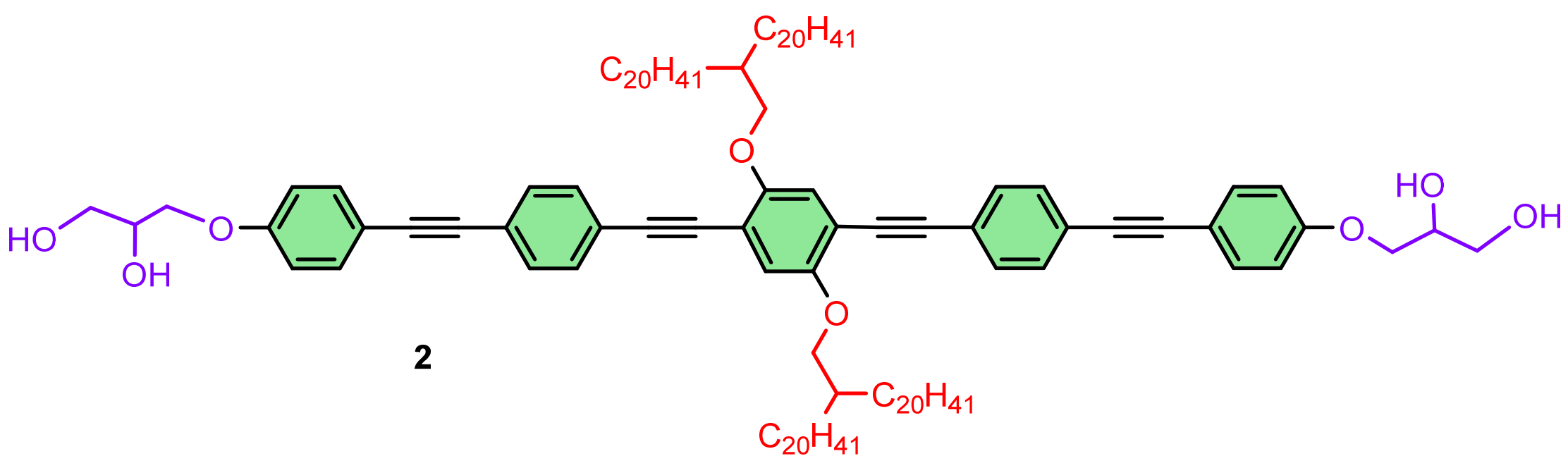

2

Figure S9. Chemical formula of compound **2**.

7. References

[S1] B. Glettner, F. Liu, X. Zeng, M. Prehm, U. Baumeister, M. Walker, M. A. Bates, P. Boesecke, G. Ungar, C. Tschierske, *Angew. Chem. Int. Ed.* **2008**, *47*, 9063-9066

[S2] S. Poppe, M. Poppe, H. Ebert, M. Prehm, C. Chen, F. Liu, S. Werner, K. Bacia, C. Tschierske, *Polymers* **2017**, *9*, 471-499.

[S3] S. Werner, H. Ebert, B.-D. Lechner, F. Lange, A. Achilles, R. Bärenwald, S. Poppe, A. Blume, K. Saalwächter, C. Tschierske, K. Bacia, *Chem. Eur. J.* **2015**, *21*, 8840-8850.

[S4] A. Immirzi, B. Perini, *Acta Crystallogr.*, **1977**, *A33*, 216-218.
